# P-768. Gene Expression Profiles for Classifying Clinical Spectrum and Monitoring Treatment Responses in Tuberculoid and Lepromatous Leprosy

**DOI:** 10.1093/ofid/ofae631.963

**Published:** 2025-01-29

**Authors:** Debdeep Mitra

**Affiliations:** Command Hospital Air Force Bangalore, Bangalore, Karnataka, India

## Abstract

**Background:**

Leprosy ranks as the second most prevalent mycobacterial disease worldwide. Various host genetic factors have been implicated in leprosy's sustained prevalence. Over the last decade, genetic epidemiology methodologies, such as genome-wide association studies (GWAS), have pinpointed over 30 loci associated with leprosy susceptibility. Nevertheless, GWAS loci typically encompass multiple genes, presenting a challenge in identifying causal candidates for each locus. Validating previously identified candidate biomarkers and discovering additional gene expression profiles are essential steps towards enhancing the diagnosis of Leprosy (Hansen's Disease) and monitoring treatment responses

**Methods:**

The study involved assessing the expression levels of 126 immune-related genes in the skin biopsy samples of 96 individuals diagnosed with Leprosy, with a positive slit skin smear for acid-fast bacilli, and 20 healthy controls without Mycobacterium leprae. This evaluation was conducted using focused gene expression profiling via dual-color Reverse-Transcription Multiplex Ligation-dependent Probe Amplification assay. Additionally, gene expression levels were monitored twelve months post-Multi-Drug Therapy in 88 Leprosy patients as part of the follow-up assessment.

**Results:**

Expression patterns of 31 host genes effectively distinguished between Pauci and multibacillary cases compared to both slit skin positive individuals and healthy controls at baseline. Notably, despite completion of multi-drug therapy at 12 months, the expression levels of most discriminating genes did not return to baseline (except for TLR10, BLK, CD38, SLAMF7, HS3ST2, and CD40LG mRNA), with full normalization observed only at the 24-month follow-up.

**Conclusion:**

Our findings suggest that comprehensive gene expression signatures are necessary to assess transcriptomic responses in skin biopsies during and after successful multi-drug therapy for Hansen’s disease. This approach is crucial for accurate diagnosis and the characterization of drug-induced relapse-free cure. It involves integrating genes that normalize completely during the 12-month treatment phase with those that only fully return to baseline levels during the post-treatment resolution phase.

**Disclosures:**

**All Authors**: No reported disclosures

